# Validation of ancillary procedures on formalin liquid fixed aspiration cytologic samples: from minimum to maximum

**DOI:** 10.1093/ajcp/aqaf117

**Published:** 2025-11-28

**Authors:** Orsolya Rideg, Tímea Dergez, Arnold Tóth, Tamás Tornóczky, Gábor Pavlovics, Endre Kálmán

**Affiliations:** Department of Pathology, Medical School and Clinical Center, University of Pécs, Pécs, Hungary; Institute of Bioanalysis, Medical School and Clinical Center, University of Pécs, Pécs, Hungary; Department of Medical Imaging, Medical School and Clinical Center, University of Pécs, Pécs, Hungary; Department of Pathology, Medical School and Clinical Center, University of Pécs, Pécs, Hungary; Department of Surgery, Medical School and Clinical Center, University of Pécs, Pécs, Hungary; Department of Pathology, Medical School and Clinical Center, University of Pécs, Pécs, Hungary

**Keywords:** ancillary technique, alternative liquid-based cytology, agarose-based cell block, formalin fixatives, fine-needle aspiration, immunohistochemistry, validation

## Abstract

**Objective:**

We sought to present an alternative liquid-based cytologic (aLBC) and an agarose-based cell block (CB) preparation method for formalin-fixed fine-needle aspirations (FNAs), using breast cancer as a model, and to perform a validation procedure for immunohistochemical (IHC) assays.

**Methods:**

Between 2024 and 2025, 18 breast cancer FNA cases were collected and processed into agarose-based CB, and by applying the dropped reaspirated monolayer dried preparation method, into aLBC. Matched formalin-fixed, paraffin-embedded surgical specimens from the same patients served as gold-standard controls. Ten immunomarkers were assessed using validated IHC protocols. Human epidermal growth factor receptor 2 (HER2) status was evaluated by both IHC and dual-probe fluorescence in situ hybridization (FISH) according to 2023 American Society of Clinical Oncology/College of American Pathologists guidelines. Staining intensity, percentage of positive cells, and marker concordance were analyzed across all specimen types. Diagnostic performance metrics were calculated, and intermethod agreement was assessed using Cohen’s κ coefficient (κ > 0.75 considered acceptable).

**Results:**

The sensitivity, specificity, and either negative or positive predictive values and the accuracy values were 100% at all the tested immunostains and at the HER2 FISH assays.

**Conclusions:**

Accurate diagnosis from scant FNA material is an increasing demand in cytopathology. The validated aLBC and CB preparation methods proved to be cost-effective, efficient for ancillary testing, and reliable for IHC, even in low-cellularity samples.

Key points

- Neutral buffered formalin fixatives are appropriate for ancillary studies.
- Validation of ancillary tests on cytologic materials bears clinical importance.
- Alternative liquid-based cytologic preparation could overcome the difficulties of low cellularity.

## INTRODUCTION

Cytopathology is one of the most rapidly developing fields in pathology.^1^^,^^2^ Based on the morphology, it mostly provides broader diagnostic categories; however, via successfully integrating ancillary techniques into routine workup, its goal is to establish more definitive diagnoses.^3‐7^ Currently, immunohistochemistry (IHC) is the most widely used ancillary tool in the pathology practice. Relying on cytomorphology and a clinical history-based, wisely selected immunopanel, IHC is essential in determining the primary site of the malignancy as well as predicting prognostic markers and response to therapy.^8^^,^^9^ Nevertheless, in certain cases, such as the subclassification of lymphomas and the determination of human epidermal growth factor receptor 2 (HER2) gene amplification status, orthogonal molecular techniques, including polymerase chain reaction (PCR)–based methods or fluorescence in situ hybridization (FISH), are needed to confirm the results of IHC.

Doing a lot with little is imperative. However, small, limited-quantity samples, especially obtained with minimally invasive procedures, such as fine-needle aspiration (FNA), could be challenging for pathologists, who must balance between extracting the maximal diagnostic information and minimizing the patient’s burden.^10‐14^

Cytologic evaluation could be performed on different specimen types, including conventional smears, cytospin, liquid-based cytology (LBC) preparations, and cell blocks (CBs), which are the most commonly used ways of cell preservation.^15‐18^

Important advantages of CBs include the increased potential for yielding multiple sections for ancillary studies, particularly for IHC, which allows storing cytologic material for future diagnostic and research purposes, and it has the potential to highlight architectural features that may not be present or easily recognized in other cytologic preparations.^13^^,^^18^^,^^19^ Over time, many CB techniques have evolved from the common and low-cost agarose-based and plasma thrombin procedures to the more time-consuming HistoGel (Epredia™ HistoGel™ Specimen Processing Gel) and collodion bag methods, leading up to the Cellient (Hologic, UK) automated method.^16^^,^^20^ The basic protocol is the same in all of them: it is a cellular concentration from liquid-based material followed by histopathologic processing.^21^ A large number of studies exist in the literature on the role of CBs, but it is difficult to compare the data because multiple factors influence the results, such as variability during the acquisition and processing of the samples.^17^^,^^21‐23^ The high sample variety and diversity in methods, as characteristics of the area, could explain why there are no standard and widely accepted, objective criteria for triaging cytology samples suitable for CB preparation and processing purposes so far.^17^^,^^19^^,^^24‐26^

The importance of liquid-based, non-CB specimens is undeniable. As these preparations usually depend on alcohol-based fixatives, they have mostly been used for morphologic diagnostics. Despite the growing demand and efforts using such materials for ancillary techniques, it can be challenging and requires great caution. In the case of IHC assays, the results could be doubtful since they are optimized for formalin-fixed, paraffin-embedded (FFPE) tissue materials.^4^^,^^24^^,^^27‐29^

Based on the report of the European Federation of Cytology Societies (EFCS), a wide variety of methods characterize the preparation of cytology samples that have been used for IHC.^30^ However, the review clearly indicates that most laboratories might also successfully use less-known alternatives for specimen preparations.^31^^,^^32^ Well-documented, standardized protocols, experiences, and published data from these laboratories could be very important in overcoming the difficulties of low-cellular, scanty cytology samples.^14^^,^^15^^,^^24^^,^^30^^,^^33^^,^^34^

Along with the increasing reliance on cytopathology, standardization regarding preanalytics (eg, sample collection, transportation, fixation, triage, handling, and storage) and recommendations for IHC assay optimization for cytology samples and validation are more important than ever.^3^^,^^14^^,^^15^^,^^29^^,^^30^^,^^34‐36^

Since most IHC protocols are validated for FFPE tissue samples, recommendations and consensus opinions of the EFCS and the College of American Pathologists (CAP) must be acknowledged. According to these, each laboratory should validate its own IHC on cytology samples and compare the results with FFPE tissue slides to ensure accuracy.^29^^,^^35‐37^ Because separate validation and optimization of all immunomarkers on all potential cytologic specimens cannot be practically accomplished and because it is impossible to obtain cytology slide controls for every possible fixation, processing mode, and specimen type, laboratories are suggested to test a selected set of frequently used markers in different cytologic specimen types in IHC assays.^35^ Markers selected for testing should be defined as nuclear, membrane, and cytoplasmic.^37^ Determining the number of positive and negative cases for testing and recognizing the possible false-negative and false-positive results due to inadequate application are the medical laboratory manager’s responsibility.^9^

Despite the growing evidence of FNA samples in the past decade, to adapt to the increased requirements and get diagnostic, prognostic, and even therapeutic information as much as possible out of the tumor content of small and limited cytology samples, our department cooperated with the Department of Medical Imaging, Medical School and Clinical Center of Pécs, about cytology material handling after acquisition.

The aim of our study was to present an alternative LBC (aLBC) and agarose-based CB preparation method for formalin-fixed FNA samples and to perform a validation procedure for immunohistochemical assays. Results of 10 immunostainings, frequently used on cytologic preparations, were compared to the gold-standard histologic sections of the same sample. The study was conducted on breast cancer specimens, as these cases meet the requirements of the validation study, providing cytologic and supposedly tissue materials as well. To confirm the HER2 immunostain results, HER2 FISH analysis of the cytologic preparations and the gold-standard histologic sections was also performed in our study.

## MATERIALS AND METHODS

The sampling was performed from January 24, 2024, to May 30, 2025. Consecutive cases were selected that provided all the sample types needed for the study. Overall, 18 breast FNA specimens taken at the Department of Medical Imaging, Medical School and Clinical Center of Pécs, as alternative liquid-based and CB preparations, were processed. In addition, at a later date, surgically removed breast cancer tissues of surgical specimens of the same patients from the Department of Surgery, Medical School and Clinical Center of Pécs were applied in the validation study, the latter providing gold-standard material. Processing of the samples, as well as the IHC and HER2 FISH assays, was accomplished at the Department of Pathology, Medical School and Clinical Center of Pécs. This study received ethical approval from the Clinical Research Ethics Committees of the University of Pécs (9916-PTE 2024).

### Smear, cell block, aLBC, and tissue preparation

Two smears were made from an ultrasound-guided fine-needle aspirate: one for conventional hematoxylin and eosin (H&E) and the other for Giemsa stainings. Liquid fixation of the aspirate was performed by rinsing the needle and the syringe in 1% neutral buffered formalin (NBF). Processing of the FNA material was performed 18 to 24 hours after sampling. To collect the cells from the 1% NBF fixative, it was centrifuged for 5 minutes at 2000 rpm at room temperature. The supernatant was removed, and a part of the cell pellet was placed on filter paper to form a cell button. Then, it was removed from the filter paper with a sharp blade and embedded in 3% agarose (REANAL; Bacteriological Agar) and transferred to a cassette for further processing as histologic specimens until paraffin embedding. The CB processing schedule is summarized in **Table 1**. The other part of the cell suspension was used in the so-called dropped reaspirated monolayer dried preparation (DReaMDP) method, forming an aLBC preparation. Namely, after placing 10 µL of the residual cell suspension on a silanized slide (Superfrost Plus Adhesion Microscope Slides; Epredia), the drop was reabsorbed, forming a spot approximately 3 mm in diameter. Routine H&E staining, the IHC assays, and the HER2 FISH analysis were scheduled after drying the slides in an incubator at 56 °C for 5 minutes.

**Table 1:** Cell Block Processing Schedule

Bottle	Solution	Temperature	Time
1.	70% IPA	RT	2 h
2.	70% IPA	RT	2 h
3.	Absolute Alcohol	RT	2 h
4.	Absolute Alcohol	RT	1.5 h
5.	IPA + Absolute Alcohol (2:1)	RT	1 h
6.	IPA + Absolute Alcohol (2:1)	RT	1 h
7.	IPA + Aceton (2:1)	RT	1 h
8.	Aceton + Xyelene (1:1)	RT	1 h
9.	Xyelene	RT	1 h
10.	Xyelene	RT	1 h
11.	Wax	60 °C	2 h
12.	Wax	60°C	1 h

Abbreviations: IPA, isopropyl alcohol; RT, room temperature.

Surgical specimens of the patients were usually available 1 to 2 months after the FNA procedure. The native breast tissues were processed within 60 minutes of removal from the patients. The sample was cut and placed into 4% NBF for 24 hours. Slices of the lesion were transferred to cassettes for further processing until paraffin embedding. Sections of the CB and the FFPE tissue were placed on the same silanized slide for H&E staining, IHC assays, and HER2 FISH analysis.

The immunopanel consisted of the following immunomarkers: HER2, estrogen receptor (ER), progesterone receptor (PR), Ki-67, cytokeratin 5 (CK5), cytokeratin 7 (CK7), GATA binding protein 3 (GATA3), P40, E-cadherin, and Ber-Epithelial antigen 4 (BerEp4) (**Table 2**). In the case of E-cadherin and BerEp4 immunostains, the IHC was carried out on the Autostainer Link 48 (DAKO), using the EnVision Flex/K8000 (Agilent) visualization system, while the other antibodies were assessed using Bond Max (Leica), applying Bond Polymer Refine Detection Kit (Leica). Immunohistochemical tests were performed using the same protocol as those validated for surgical materials.

**Table 2:** Antibodies Verified for Use in Immunohistochemistry

Antibody	Clone (manufacturer)	Dilution	Localization
HER2 (c-erbB-2)	Rabbit Monoclonal SP3 (Histopathology Ltd)	1:150	Membrane
Progesterone receptor (PR; breast)	Rabbit Monoclonal SP2 (Histopathology Ltd)	1:100	Nuclear
Estrogen receptor (ER; breast)	Rabbit Monoclonal SP1 (Histopathology Ltd)	1:50	Nuclear
Ki-67 (Mib1)	Mouse Monoclonal B56 (Histopathology Ltd)	1:200	Nuclear
GATA 3	Mouse Monoclonal L50-823 (Biocare Medical)	1:150	Nuclear
P40	Mouse Monoclonal BC28 (Biocare Medical)	1:50	Nuclear
CK7	Mouse Monoclonal 12/30 (Agilent; DAKO)	1:3000	Cytoplasmic
CK5	Mouse Monoclonal NCL-L-CK5 (Leica)	1:150	Cytoplasmic
E-cadherin	Mouse Monoclonal NCH-38 (Agilent; DAKO)	1:400	Membrane
Ber-Epithelial antigen 4	Mouse Monoclonal BerEP4 (Agilent; DAKO)	1:100	Cytoplasmic

The cytologic preparations and the histologic materials themselves served as positive and negative controls; the negative controls were the resident benign cell populations and nonreactive malignant cells.

### Aspects of evaluating IHC

Distinguishing the cytoplasmic and membrane HER2 immunosignals in aLBC preparation to characterize the HER2-low type is often unreliable. To overcome these difficulties, the scoring of the samples was the following: 0 for negative and/or 1× negative and 1 for positive/FISH indicated. However, to follow the American Society of Clinical Oncology (ASCO)/CAP guidelines, HER2 status of the histologic and CB preparations was graded as 3+ (strong and diffuse), 2+ (moderate and diffuse), 1+ (focal and weak), and 0 (negative).^38‐40^ The PR and ER proportions of expression (PS) and intensity (IS) were calculated based on the Allred score guideline.^41^ The Ki-67 index was determined based on the St Gallen 2013 consensus; high proliferation was defined as a cutoff of a Ki-67 level of 20% or more, low proliferation as a Ki-67 level of less than 20%, and negative as 0.^38‐42^^,^^43^ For the other immunomarkers (CK5, CK7, P40, E-cadherin, GATA3, BerEp4), the percentage of positive cells was scored at quarter intervals (0%, 1%-25%, 26%-50%, 51%-75%, 76%-100%). The intensity of staining compared to control FFPE tissue was scored on cytology materials using a binary method as follows: cytology equal to or greater than FFPE (score 1) and cytology less than FFPE (score <1).

Two specialized pathologists (EK, TT) assessed IHC; if the evaluations were different, the discrepancy was discussed. Across all 540 marker evaluations by 2 independent observers (EK, TT), minor differences in the quantity of positive-stained cells were observed in 10 cases, corresponding to an observed agreement of 98.1%. The calculated Cohen’s κ value was 0.96, indicating almost perfect agreement between observers.

All slides were assessed using a conventional light microscope. For CB and aLBC slides, a minimum of 100 lesional cells was considered adequate.

### HER2 FISH analysis

HER2 FISH testing was performed in all cases, applying the in vitro diagnostic regulations-certificated dual-probe HER2 FISH assay, using probes for the HER2 (ERBB2) and the α-centromeric region of chromosome 17 (CEP17) (MetaSystems XL ERBB2 [HER2/NEU] Amplification Probe); for fluorescent nuclear counterstaining, we used DAPI (Cytocell; DAPI Antifrade ES; DES150L). Optimization of combined heat and enzymatic nucleic acid retrieval steps and internal validation were previously performed in our laboratory using aLBC preparation and agarose-based CB. HER2 FISH results were interpreted using the National Consensus Statement based on the updated 2023 ASCO/CAP guidelines. HER2 FISH was regarded as amplified if the HER2/CE17 ratio was 2.0 or higher, at a minimum of 50 cells counted/preparation.

### Statistical analysis

All statistical analyses were conducted using the IBM SPSS 29 software. The total scores were used as a basis for comparing and evaluating the negative and positive results. The sensitivity, specificity, and negative and positive predictive values were determined through CB and aLBC analyses when compared with FFPE as a control.

To evaluate the results for progesterone and estrogen receptors and for the HER2 FISH assay, we calculated Cohen’s κ coefficient. This statistic is employed to assess interrater reliability for qualitative (categorical) variables, indicating the extent of agreement between the 2 methods. The κ value quantifies the observed agreement in comparison to the expected agreement, with a maximum value of 1, signifying perfect agreement. The level of agreement between the 2 evaluations was deemed acceptable if κ > 0.75.

Interobserver agreement for intensity and the proportion of expression scores was evaluated using Cohen’s κ. To address potential prevalence and bias effects due to highly skewed distributions, we additionally calculated prevalence- and bias-adjusted κ (PABAK) and Gwet’s AC1. For discordance rates in small samples, we computed 95% Clopper-Pearson exact confidence intervals to estimate the uncertainty around observed agreement. These metrics provide complementary information on the reliability and reproducibility of the staining evaluations.

## RESULTS

**Figure 1:**
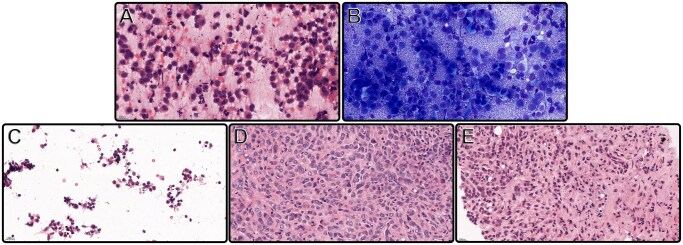
Cytology smears of an invasive breast cancer of no special type, hematoxylin and eosin (H&E) stained (**A**) (400×) and Giemsa stained (**B**) (400×). Equal intensity and proportional H&E staining of lobular carcinoma breast cancer subtype cells: alternative liquid-based cytology preparation (**C**) (400×); formalin-fixed, paraffin-embedded tissue section (**D**) (400×); and agarose-fixed cell block section (**E**) (400×).

Morphologic analysis and quality control of the materials were done by assessing the H&E- and Giemsa-stained smears, the H&E-stained tissue and CB sections, and the aLBC preparation of the samples (**Figure 1**). Based on the microscopic examination, all cases were affirmed to be appropriate for further IHC analysis.

All the FFPE and cytologic preparations gave meaningful results to the 10 immunostains tested. Neither false-positive nor false-negative results occurred during the analysis. In all cases, the FFPE and cytologic preparations gave equal quality (positive or negative) results, with no variance. **Table 3** summarizes the subtypes of the 18 breast cancer cases tested, presenting the number of positive and negative ones on each immunostain. The prevalence of the positive immunomarkers was consistent with what is usual in breast cancer: 100% of the samples were Ki-67 and GATA 3 positive; 94.4% were BerEP4 positive; 83.3% were E-cadherin and ER positive; CK7 and PR occurred at 72.2% and 55.5%, respectively; 16.6% of the samples were HER2 and P40; and 11.1% were CK5 positive.

**Table 3:** Number of Positive and Negative Cases Regarding Breast Cancer Subtypes and Immunostains

Antibody	Invasive breast cancer of no special type (n = 12)		Metaplastic carcinoma (n = 1)		Invasive micropapillary carcinoma of breast (n = 1)		Infiltrating duct carcinoma + lobular carcinoma (n = 1)		Lobular carcinoma (n = 3)			
	+	–	+	–	+	–	+	–	+		–	
HER2 (c-erbB-2)	3	9	0	1	0	1	0	1	0		3	
Progesterone receptor (PR; breast)	7	5	0	1	1	0	0	1	2			1
Estrogen receptor (ER; breast)	10	2	0	1	1	0	1	0	3	0		
Ki-67 (Mib1)	12	0	1	0	1	0	1	0	3			0
GATA 3	12	0	1	0	1	0	1	0	3			0
P40	2	10	1	0	0	1	0	1	0			3
CK7	11	1	1	0	1	0	0	1	3			0
CK5	1	11	1	0	0	1	0	1	0			3
E-cadherin	12	0	1	0	1	0	1	0	0			3
Ber-Epithelial antigen 4 (BerEP4)	12	0	1	0	1	0	1	0	2			1

Abbreviations: +, positive case; –, negative case.

During the analysis, positive and negative results were scored, classified, and subsequently compared and evaluated. According to these, both the sensitivity and the specificity, as well as the negative and positive predictive values, were 100% when comparing both the CB and the aLBC preparation to the gold-standard FFPE tissue control. The accuracy values were also 100%.

Based on our detailed quantitative grading systems, when the immunostaining results of the CBs and the aLBC preparations were compared to the FFPE tissue gold standard, no differences were found in the amount of positive cells regarding HER2, CK7, Ki-67, P40, and GATA3 (**Table 4**). In 1 of the 2 CK5-positive cases, both cytologic preparations displayed signals that were weaker by 2 quartiles. For E-cadherin, in 1 of the 15 positive cases, both the aLBC and CB exhibited a signal that was weaker by three quartiles when compared to the tissue section. In the case of BerE4, the signal was one quartile lower in 1 of the 17 positive aLBC preparations compared to the tissue section (**Table 4**).

**Table 4:** Immunohistochemical Studies of the 18 Breast Cancer Specimens^a^

Antibody (source)	Quantitative difference from FFPE (No. of cases)	Intensity difference from FFPE (No. of cases)
HER2 (c-erbB-2)	0 (18)	1 (18)
CK5	0 (17); 1 (–1 CB, –1 aLBC)	1 (18)
CK7	0 (18)	1 (18)
Ki-67 (Mib1)	0 (18)	1 (18)
P40	0 (18)	1 (18)
E-cadherin	0 (17); 1 (–1 CB, –1 aLBC)	1 (18)
GATA3	0 (18)	1 (18)
Epithelial antigen 4 (BerEP4)	0 (17); 1 (–1 aLBC)	1 (18)

Abbreviations: aLBC, alternative liquid-based cytopreparation; CB, cell block; FFPE, formalin-fixed, paraffin-embedded.

a 0 shows the equality between FFPE, CB, and aLBC; 1 shows the negative difference from FFPE regarding CB and aLBC.

**Figure 2:**
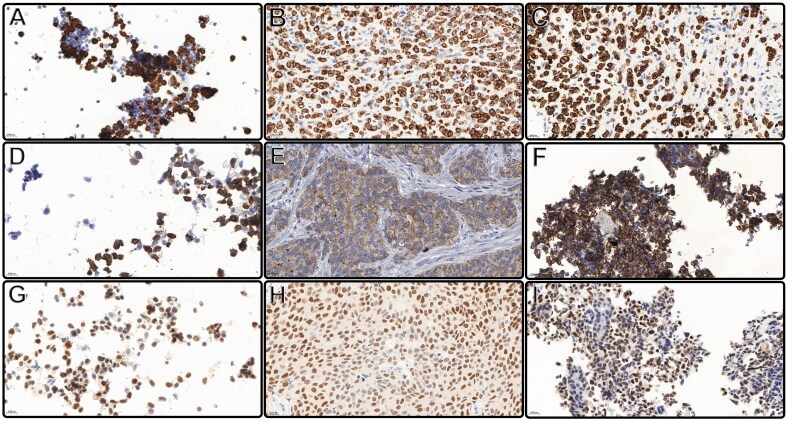
Equal intensity and proportional. CK7 cytoplasmic staining of lobular carcinoma breast cancer subtype cells: alternative liquid-based cytology (LBC) preparation (**A**) (400×); formalin-fixed, paraffin-embedded (FFPE) tissue section (**B**) (400×); and agarose-fixed cell block (CB) section (**C**) (400×). E-cadherin membrane staining of invasive breast cancer of no special type cells: alternative LBC preparation (**D**) (400×), FFPE tissue section (**E**) (400×), and agarose-fixed CB section (**F**) (400×). GATA3 nuclear staining of invasive breast cancer of no special type cells: alternative LBC preparation (**G**) (400×), FFPE tissue section (**H**) (400×), and agarose-fixed CB section (**I**) (400×).

In addition, the quality and intensity of the stainings were the same in all cases for all sample types (**Table 4**). **Figure 2** presents positive immunostains of 3 different immunolocalization markers: CK7 as cytoplasmic, GATA3 as nuclear, and E-cadherin as a membrane marker.

**Figure 3:**
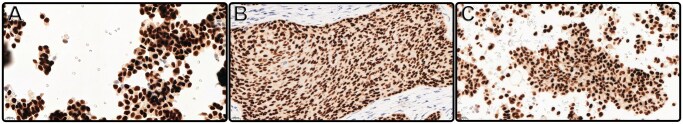
Equal intensity and proportional estrogen receptor nuclear staining of invasive micropapillary carcinoma of breast subtype cells: alternative liquid-based cytology preparation (**A**) (400×); formalin-fixed, paraffin-embedded tissue section (**B**) (400×); and agarose-fixed cell block section (**C**) (400×).

In the case of the PR and ER, Cohen’s κ coefficient was determined (**Table 5**). According to the data, the PR showed very high similarity in the different samples (κ =  1.00, IS FFPE vs IS CB, F06BF03DS; κ = 0.902, FFPE vs IS aLBC and IS CB vs IS aLBC; κ =  0.841, PS FFPE vs PS CB and PS aLBC; κ =  0.922, PS CB vs PS aLBC). In relation to the ER analysis, the κ value was 1 in all comparisons (**Figure 3**).

**Table 5:** Comparing the agreement of Progesterone and Estrogen Receptor Results

Antibody (source)	Comparisons	κ value
Progesterone receptor (PR; breast)	IS FFPE vs IS CB IS FFPE vs IS aLBC IS CB vs IS aLBC PS FFPE vs PS CB PS FFPE vs PS aLBC PS CB vs PS aLBC	1.00 0.902 0.902 0.841 0.841 0.922
Estrogen receptor (ER; breast)	IS FFPE vs IS CB IS FFPE vs IS aLBC IS CB vs IS aLBC PS FFPE vs PS CB PS FFPE vs PS aLBC PS CB vs PS aLBC	1.00 1.00 1.00 1.00 1.00 1.00

Abbreviations: aLBC, alternative liquid-based cytopreparation; CB, cell block; FFPE, formalin-fixed, paraffin-embedded; IS, intensity of staining; PS, positivity of staining.

The structure of the CB made it suitable for classifying the HER2 grade in all cases, and its comparison to the FFPE control was based on ASCO/CAP guidelines. According to these, 14 samples were HER2 0 negative, 1 sample was 1+ negative, and 3 samples were 3+ positive. Based on the results, sensitivity, specificity, negative and positive predictive values, and accuracy were 100%.

**Figure 4:**
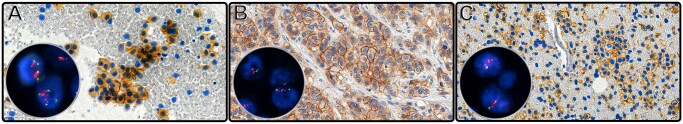
Equal intensity and proportional human epidermal growth factor receptor 2 membrane staining of invasive breast cancer of no special type cells and confirmation with the corresponding HER2 fluorescence in situ hybridization assay: alternative liquid-based cytology preparation (**A**) (400×); formalin-fixed, paraffin-embedded tissue section (**B**) (400×); and agarose-fixed cell block section (**C**) (400×).

HER2 FISH results of the cytologic preparations and the corresponding surgical specimens showed perfect correlation (κ = 1) with either the HER2 immunostaining results or pairwise assessment. Of the 18 samples, 15 appeared HER2 FISH negative (class 5), while the 3 HER2 immunopositive samples were also HER2 FISH amplified (class 1) in all tested materials (**Figure 4**). The sensitivity, specificity, and negative and positive predictive values were 100%.

## DISCUSSION

Immunohistochemistry is progressively becoming an integral and necessary part of routine cytopathology diagnostics. It highlights the utility of CB and liquid-based cytopreparations as excellent sources of the assay and the importance of well-defined workflows.^44‐46^ Although IHC is a well-accepted method for cytopreparations, the lack of procedure adjustment and validation makes the accuracy of the assay and interpretation of the results doubtful, leading to confusion in many laboratories.^47^

In our study, using 1% NBF-based liquid fixatives of breast FNA, we successfully established an alternative liquid-based cytologic and agarose-based CB preparation procedure and validated 10 frequently used immunomarkers on them, comparing the results to the gold-standard FFPE histology material. Both presented preparation techniques are cost- and time-effective, reproducible, and can provide multiple slides, making them easily integrable into the routine workflow. In addition, in both cytologic preparations, the cells are concentrated in small-diameter round areas, making the evaluation easier.

The main cause of dissatisfaction with the CB preparation, on the one hand, remains the low cellularity. Based on our experiences and according to the literature, at least 50 000 cells are required for preparing a CB bearing clinical utility.^24^ In cases with a high rate of unicellular dispersion and a low cell count, the presented DReaMDP method produced aLBC, which ensures an effective choice, while as low as 1000 to 3000 cells could provide appropriate material for ancillary techniques. However, it must be highlighted that quality and contexts often rule over the importance of cell amount,^32^ [Supplement Table 1].

On the other hand, different fixatives, especially alcohol or alcohol-based solutions, such as PreservCyt, Cytolyt, CytoRich Red, and SurePath, used in LBC and Cellient CB, are known to cause variable and often significantly lower staining of different antibodies, even antibody clones, compared to formalin-based materials.^48‐50^ Indeed, referring to the literature, alcohol lacks the immunostaining of S100 and hormone receptors, resulting in possible false negativity.^16^^,^^35^

As supported by our findings and the cited literature, to avoid false negatives and ensure accuracy, unless using formalin fixation, each antibody (possibly antibody clone) must be validated for the fixation method.

Although the 10 immunomarkers tested are important in breast cancer diagnostics, CK5, HER2, and P40 had a low prevalence (11.1%, 16.6%, and 16.6%, respectively) in our study; however, these results are in line with the published data.^51‐57^ While we acknowledge that the cohort remains limited, particularly for low-prevalence markers, to emphasize the reliability of the cytopreparation methods, we also confirmed our data by applying prevalence-adjusted agreement measures, which proved high concordance (PABAK = 0.889, Gwet’s AC1 = 0.941), with a 95% Clopper-Pearson confidence interval for the discordance rate in the 18 cases of 0 to 0.185. The narrow Clopper-Pearson confidence intervals observed in our analysis indicate that the agreement estimates are stable and are likely to remain at a similarly high level in larger sample sets. The other 7 markers appeared positive at 55.5% to 100%.^56^^,^^57^

The outcome of our study proved the utility of the presented methods. Neither false negativity nor false positivity occurred during the analysis. Based on the results, we can declare that sensitivity, specificity, and the negative and positive predictive values were 100% when comparing the CB and the alternative LBC preparations to the FFPE tissue control. Additionally, the accuracy values were also 100% in both cases.

The quantities of positive cells, regarding HER2, CK7, Ki-67, P40, and GATA3, were consistent in all cases in all sample materials. With E-cadherin staining, in 1 patient, both cytologic preparations presented a lower positive cell count when compared to the FFPE tissue control. In the case of the BerEp4 antigen, the LBC material was weaker than the FFPE section on only 1 occasion. The possible explanation could be the loss of material during the cytologic process. In addition, a lower positive cell count was observed at CK5 staining on 1 occasion for both cytologic preparations, probably due to the high heterogeneity distribution of the CK5-positive cells in the sample. However, despite the small differences, the intensity of the immunostainings was the same on all occasions.

Based on the All Red Scores data, the Cohen’s κ coefficient of PR and ER was determined.

For progesterone, Cohen’s κ for IS was 1.0 for FFPE vs CB, while minor deviations (<1.0) were observed for FFPE vs aLBC and CB vs aLBC; κ for PS also showed small deviations (<1.0) for both CB and aLBC. Importantly, these differences did not alter the categorical positive/negative status.

The Cohen’s κ for ER was 1 in all comparisons.

Based on our comparative analysis of numerous antibodies, it can be concluded that those IHC assays previously validated for surgical specimens are also suitable for cytologic preparations without any changes.

According to international guidelines, markers such as ER, PR, HER2, and Ki-67, which are used to determine biological behavior or known as therapeutic targets, should be analyzed by IHC on the resected primary tumor or a preoperative core biopsy specimen.^40^^,^^43^^,^^58^ Despite the protocol, FNA is sometimes the only choice to obtain tumor cells from metastatic lesions. In addition, in some cases, immunohistochemical assessment of a breast tumor is performed on cytologic material, even though a reevaluation on histologic material follows. In such cases, the above recommended workflow could provide a useful solution for setting up accurate pathologic diagnoses, shorten medical decision time, and/or assist in further examinations (eg, next-generation sequencing, FISH).

In accordance with the literature, the results of HER2 FISH analysis showed a perfect correlation with the HER2 immunostain results or in pairwise assessment when comparing the cytologic preparations to tissue sections.^59‐61^

It is the collective liability of the clinician, radiologist, and cytopathologist to provide the patient with a prompt diagnosis and adequate medical assistance as quickly as possible and without additional intervention. On the one hand, obtaining the sample, having clear communication between the involved parties, using image-guided procedures, and optimizing the techniques are essential aspects to achieve good quality and a sufficient amount of material for the analysis.^62‐65^ On the other hand, well-trained cytopathologists and cytotechnologists are needed for the whole procedure. They should be comfortable with triaging cytology specimens and be familiar with the wise use of samples and the correct interpretation of ancillary test results.^2^^,^^61^ Finally, clinicopathologic correlation is critical to filter out false-negative and false-positive results.^2^^,^^65^

Beyond the detailed validation study, our department has well over a decade of experience reliably adapting IHC and HER2 FISH assays on formalin-fixed aLBC preparations and on CBs. Besides the conventional assessment of around 3800 cytologic materials received in the past year, we also used the submitted cytopreparations and IHC assays in about 1220 (32%) cases, either to set up or verify the diagnosis. In addition, around 90 HER2 FISH analyses were performed.

The validation study above followed the CAP guidelines, and we are continuously extending it to more immunomarkers.

## CONCLUSION

This study demonstrates that formalin-fixed aLBC and agarose-based CB preparations are reliable substrates for IHC and HER2 FISH analysis in breast cancer FNAs. Both methods showed complete concordance with matched FFPE tissue in terms of staining quality, marker expression, and diagnostic accuracy. These validated protocols offer practical, reproducible, and cost-effective options for ancillary testing in cytopathology, especially when tissue samples are limited or unavailable. Their integration into routine diagnostic workflows can enhance the diagnostic yield of cytologic specimens and support timely clinical decision-making.

## Supplementary Material

aqaf117_Supplementary_Data

## Data Availability

The data underlying this article are available in the article and in its online supplementary material.
