# Supplementary figures and images for: Validation of ancillary procedures on formalin liquid fixed aspiration cytologic samples: from minimum to maximum
Source: Am J Clin Pathol. 2025 Nov 28;164(6):924–32. doi: 10.1093/ajcp/aqaf117 (PMC12782306; doi:10.1093/ajcp/aqaf117)

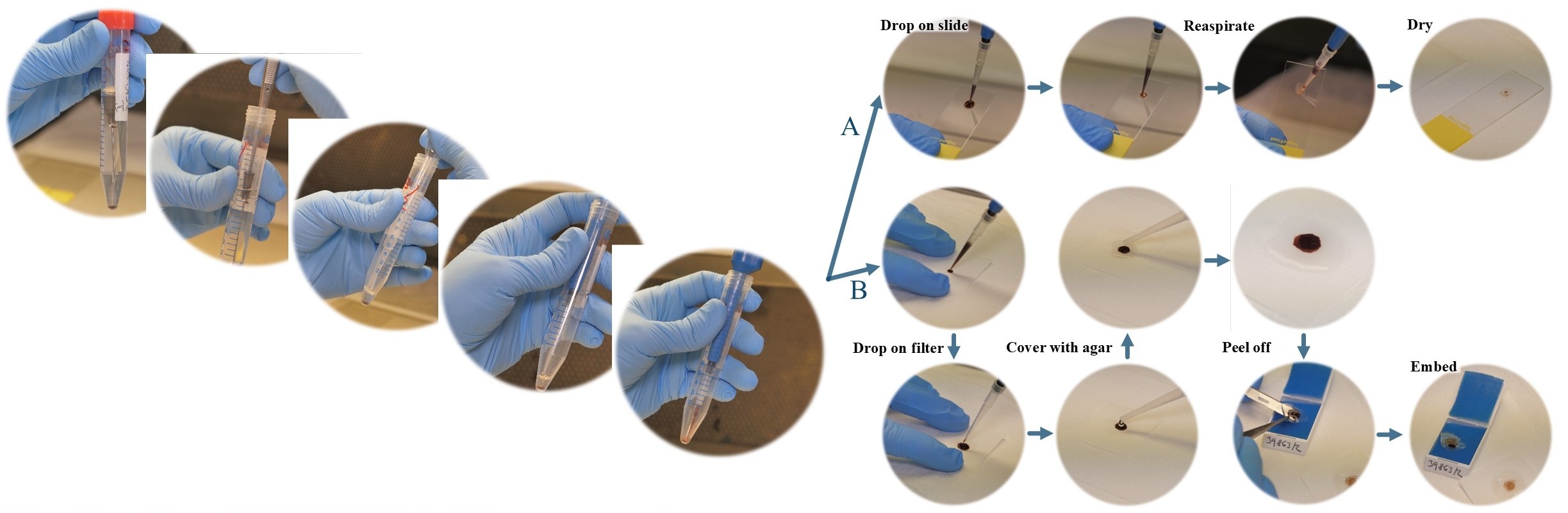

Supplement: aqaf117_Supplementary_Data [file aqaf117_supplementary_data.zip › aqaf117_Supplementary_Data/ajcp-2025-07-0385-File011.jpg]
